# IQGAP3 promotes the progression of glioma as an immune and prognostic marker

**DOI:** 10.32604/or.2023.046712

**Published:** 2024-03-20

**Authors:** XIAOFENG GAO, JUANJUAN GE, XUZHENG GAO, NA MEI, YANTING SU, SHIGANG SHAN, WENBIN QIAN, JIANGHENG GUAN, ZHENWANG ZHANG, LONG WANG

**Affiliations:** 1 Hubei Provincial Key Laboratory of Diabetic Cardiovascular Diseases, Xianning Medical College, Hubei University of Science and Technology, Xianning, 437100, China; 2 School of Stomatology and Ophthalmology, Xianning Medical College, Hubei University of Science and Technology, Xianning, 437100, China; 3 School of Basic Medical Sciences, Xianning Medical College, Hubei University of Science and Technology, Hubei University of Science and Technology, Xianning, 437100, China; 4 School of Pharmacy, Xianning Medical College, Hubei University of Science and Technology, Xianning, 437100, China; 5 Department of Neurosurgery, The General Hospital of Chinese PLA Central Theater Command, Wuhan, 430070, China

**Keywords:** IQGAP3, Tumor immune infiltration, Prognosis, Glioma, Biomarker

## Abstract

**Background:** IQGAP3 plays a crucial role in regulating cell proliferation, division, and cytoskeletal organization. Abnormal expression of IQGAP3 has been linked to various tumors, but its function in glioma is not well understood. **Methods:** Various methods, including genetic differential analysis, single-cell analysis, ROC curve analysis, Cox regression, Kaplan-Meier analysis, and enrichment analysis, were employed to analyze the expression patterns, diagnostic potential, prognostic implications, and biological processes involving IQGAP3 in normal and tumor tissues. The impact of IQGAP3 on immune infiltration and the immune microenvironment in gliomas was evaluated using immunofluorescence. Additionally, the cBioPortal database was used to analyze copy number variations and mutation sites of IQGAP3. Experimental validation was also performed to assess the effects of IQGAP3 on glioma cells and explore underlying mechanisms. **Results:** High IQGAP3 expression in gliomas is associated with an unfavorable prognosis, particularly in wild-type IDH and 1p/19q non-codeleted gliomas. Enrichment analysis revealed that IQGAP3 is involved in regulating the cell cycle, PI3K/AKT signaling, p53 signaling, and PLK1-related pathways. Furthermore, IQGAP3 expression may be closely related to the immunosuppressive microenvironment of glioblastoma. BRD-K88742110 and LY-303511 are potential drugs for targeting IQGAP3 in anti-glioma therapy. *In vitro* experiments showed that downregulation of IQGAP3 inhibits the proliferation and migration of glioma cells, with the PLK1/PI3K/AKT pathway potentially playing a crucial role in IQGAP3-mediated glioma progression. **Conclusion:** IQGAP3 shows promise as a valuable biomarker for diagnosis, prognosis, and immunotherapeutic strategies in gliomas.

## Introduction

Glioma (GBMLGG), which includes glioblastoma (GBM) and low-grade glioma (LGG), is the predominant malignant tumor originating in the central nervous system (CNS), collectively constituting approximately 80% of malignant intracranial neoplasms [1,2]. GBM stands out as one of the most formidable and persistently recurring malignant solid tumors. It accounts for 57% of the entirety of gliomas and encompasses 48% of primary malignancies within the CNS. This neoplasm is distinguished by its rapid growth, pronounced invasiveness, and notable migratory capabilities [3]. Current standard treatment approaches for gliomas primarily focus on maximal safe surgical resection, followed by adjuvant radiotherapy and chemotherapy, yet the high mortality and disability rates remain the main challenges in glioma treatment [4]. In recent years, biological targeting and immunotherapy have become hotspots in cancer treatment [5]. For instance, favorable clinical outcomes have been observed in adult patients with low-grade gliomas harboring IDH mutations and 1p/19q co-deletion. Additionally, the co-deletion of 1p/19q has been shown to predict sensitivity to temozolomide treatment [6]. Moreover, in pediatric patients, H3F3A alterations have been identified as crucial biomarkers predicting poorer outcomes [7]. Nonetheless, owing to the inherent heterogeneity and intricacy of the tumor microenvironment, coupled with the emergence of resistance to targeted therapies and immunomodulation in select patients, the management of gliomas persists as a formidable challenge. Therefore, to achieve personalized precision therapy, there exists an imperative requirement to comprehensively investigate the pivotal molecular alterations and mechanisms intricately linked with the progression of gliomas, aiming to develop novel therapeutic strategies for this deadly malignancy.

The GTPase isoleucine-glutamine sequence activator protein (IQGAP) constitutes an evolutionarily conserved gene family encompassing three distinct members: IQGAP1, IQGAP2, and IQGAP3. The family members contain the calmodulin homology domain (CHD), polyproline binding domain (WW), calmodulin-binding protein (IQ), and GTPase-activating protein-related domain (RGD). IQGAP assumes a pivotal role in intracellular signal transduction, cell proliferation, cell migration, and cell division [8–10]. Notably, within this group, IQGAP3 specifically serves an indispensable function in both the advancement of the cell cycle and the preservation of genome stability [11]. Extensive evidence substantiates the involvement of IQGAP3 in the inception and progression of diverse malignant neoplasms, with notable prominence in breast cancer, clear cell renal cell carcinoma, and lung cancer [12–14]. Furthermore, IQGAP3 significantly participates in assorted tumor-related signaling pathways, including the EGFR/ERK signaling pathway [15], ERK1/2 signaling pathway [16], MEK/ERK, and p38 signaling pathways [17]. Nonetheless, the function of IQGAP3 in gliomas remains unclear.

This study provides a comprehensive analysis of IQGAP3 in gliomas based on multi-omics data and *in vitro* experiments. We leveraged bioinformatics methodologies in conjunction with numerous publicly available databases to unravel the potential oncogenic significance of IQGAP3 in gliomas, including its expression, diagnostic, prognostic, genetic alterations, immune cell infiltration, and targeted drugs. By conducting single-gene differential analysis and correlation assessment, the cross-gene was identified, followed by enrichment analysis to identify the biological processes associated with IQGAP3. Additionally, experimental results demonstrated that IQGAP3 influences glioma cell proliferation, invasion, and migration, and is involved in various pathways in glioma development. In conclusion, the roles of IQGAP3 in immune therapy and targeted treatment may provide valuable insights for future glioma therapies.

## Materials and Methods

### Data sources

The mRNA sequencing expression data encompassing 689 glioma samples (523 LGG and 116 GBM) and 1152 corresponding normal tissue samples were acquired from TCGA (https://portal.gdc.cancer.gov/) and GTEx (https://commonfund.nih.gov/GTEx) databases, along with pertinent clinical patient information. All the downloaded data were subjected to standardization and log2 transformation. These datasets served as the foundation for assessing IQGAP3 expression levels in both normal and tumorous tissues. Furthermore, the prognostic relevance of IQGAP3 was discerned through Cox regression analysis of the patient dataset.

### Expression analysis of IQGAP3

To assess discrepancies in mRNA expression of IQGAP3 between glioma and normal tissue samples, and to explore the potential correlations of IQGAP3 expression with WHO grade, IDH mutation, and 1p/19q co-deletion, the data analysis was conducted employing R software (version 4.0.2). A significance threshold of *p*-value < 0.05 was adopted for screening, and for effective visualization, the “ggplot2” R package was employed. For the purpose of scrutinizing IQGAP3’s differential protein expression, immunohistochemical images of glioma tissues and normal brain cortex tissues were obtained from the Human Protein Atlas (HPA) database (https://www.proteinatlas.org/). Furthermore, the single-cell dataset GSE182109 was obtained through the Single-Cell Portal (https://singlecell.broadinstitute.org/single_cell). Initially, glioma was searched in the “study” section, followed by inputting “IQGAP3” to assess the expression levels of IQGAP3 in various cell types within glioma.

### Diagnostic performance analysis of IQGAP3

For the assessment of IQGAP3’s diagnostic precision, the receiver operating characteristic (ROC) curve analysis was executed utilizing the “pROC” package, with subsequent presentation of outcomes accomplished via the “ggplot2” R package. The ROC curve inherently provides a comprehensive gauge of sensitivity and specificity for continuous variables. Within this framework, the area under the curve (AUC) spans from 0.5 to 1, with an AUC above 0.9 indicating high accuracy.

### Prognostic relationship analysis of IQGAP3 for survival

In order to scrutinize the connection between IQGAP3 expression levels and the prognosis of glioma patients, the cohort was stratified into two groups based on the median IQGAP3 expression value–namely, the high IQGAP3 expression group and the low IQGAP3 expression group. To comprehensively assess the prognostic implications, three distinct prognostic parameters were compiled: overall survival (OS), disease-specific survival (DSS), and progression-free interval (PFI). Subsequently, an amalgamation of univariate and multivariate Cox regression models and Kaplan-Meier curves was employed to evaluate the prognostic significance of IQGAP3 within these specific prognostic contexts within the glioma population. For the appraisal of the impact of IQGAP3 expression on glioma patient survival, Kaplan-Meier analysis was employed. Moreover, both univariate and multivariate Cox regression analyses were executed to ascertain the autonomous prognostic value of IQGAP3 within the glioma patient cohort. The log-rank test was leveraged to compute *p*-values, accompanied by the calculation of hazard ratios (HRs) replete with their corresponding 95% confidence intervals (95% CIs). Survival analysis and subsequent visualization were undertaken utilizing R version 4.0.2. The “survival” package was harnessed for conducting survival analysis and Cox regression analyses, while the “survminer” package facilitated the graphical representation of these findings.

### Analysis of genetic modifications

We explored the genetic alterations of IQGAP3 using the online cBioPortal database (https://www.cbioportal.org/). In the “Cancer Type Summary” module of the TCGA tumor dataset, copy number alterations (CNAs) and mutation sites of IQGAP3 were observed in glioma. Furthermore, somatic mutation data were extracted from the TCGA database. Subsequently, the R package “maftools” was harnessed to render a visual depiction of the mutational landscape pertaining to the top 15 genes characterized by the highest mutation frequencies.

### The relationship between IQGAP3 expression and tumor immune infiltration

To assess the impact of IQGAP3 on immune infiltration in glioma, a single-sample gene set enrichment analysis (ssGSEA) was executed using the R GSVA package. This allowed the quantification of infiltration levels pertaining to 25 distinct immune cell types. To ascertain the connection between IQGAP3 expression and each immune cell infiltration level, Spearman’s rank correlation test was performed, and the outcomes were then effectively illustrated through the utilization of the “ggplot2” R package. Employing the ESTIMATE algorithm, an evaluation was conducted to explore the interplay between IQGAP3 expression and pertinent scores such as ESTIMATE score, immune score, and stromal score. In the context of scrutinizing the relationship between IQGAP3 and immune checkpoints, Spearman analysis was applied to gauge the correlation between IQGAP3 and pivotal molecules affiliated with immune checkpoints. To provide a visual representation of these findings, the “circlize” R package was employed. Furthermore, a comprehensive examination was extended to explore the intricate association between IQGAP3 and immune checkpoint genes, encompassing both inhibitory (24 genes) and stimulatory (36 genes) categories, as well as genes encoding immune inhibitors, chemokines, and chemokine receptor proteins. The visualization of these results was facilitated through utilization of the “ggplot2” package.

### Sample source

This study included eight glioblastoma patients who underwent surgical treatment in the Department of Neurosurgery at Hubei University of Science and Technology Affiliated Hospital from March 2023 to July 2023. None of the patients had received radiotherapy or chemotherapy prior to surgery. During the surgery, efforts were made to achieve maximal tumor resection while preserving neurological function. Subsequently, tumor tissue specimens were obtained from all eight patients and corresponding adjacent non-tumor tissues. This study was approved by the Ethics Committee of Hubei University of Science and Technology Affiliated Hospital, and informed consent was obtained from all participants or their legal guardians.

### Multicolor fluorescence immunohistochemistry (mIHC)

The surgically excised tumor and adjacent tissue were fixed in formalin for 48 h, dehydrated, embedded in paraffin blocks, and then cut into tissue sections with a thickness of 4 μm. Deparaffinization and rehydration were carried out using conventional methods. The tissue sections were placed in EDTA buffer (pH 9.0) and subjected to heat-induced antigen retrieval in a microwave oven. Subsequently, the sections were blocked with 5% bovine serum albumin (BSA) for 1 h. The sections were then sequentially stained with primary antibodies: IQGAP3 (DF4389, Affinity, China), CCR3 (GB112453, Servicebio, China), CD39 (GB111582, Servicebio, China), and CD10 (GB114689, Servicebio, China). The primary antibodies were incubated overnight at 4°C on a shaker. After washing with PBS, appropriate fluorescent secondary antibodies were applied and incubated at room temperature for 1 h: HRP-conjugated goat anti-rabbit IgG (CCR3, CD39, CD10) and Alexa Fluor 594-labeled goat anti-rabbit IgG (IQGAP3). Antigen retrieval was performed using heat fixation and tyramide signal amplification (TSA), followed by antigen retrieval through heat induction and cooling. Finally, the cell nuclei were counterstained with DAPI (G1012, Servicebio, China) and incubated at room temperature in the dark for 10 min, followed by blocking with a tissue autofluorescence quencher (G1221, Servicebio, China). Whole-slide brightfield images were acquired using a scanner (Pannoramic MIDI, 3DHISTECH, Hungary), and fluorescence images were collected using an upright fluorescence microscope (Nikon Eclipse C1, Nikon, Japan).

### Screening and enrichment analysis of IQGAP3-related differential genes

We employed the DESeq2 package within the R software environment to discern differentially expressed genes (DEGs) (|logFC| > 1, adjust *p* < 0.05) existing between the high and low IQGAP3 expression groups. The classification of these groups was based on the median IQGAP3 expression value derived from the TCGA database. Furthermore, we performed a Pearson correlation test to extract genes associated with IQGAP3 expression in glioma (setting |cor| > 0.5, adjust *p* < 0.05). Venn analysis was applied to identify the differentially expressed genes (RDEGs) that were correlated with IQGAP3 in glioma, and the ComplexHeatmap package was used for visualization.

In order to delve into plausible signaling pathways and biological functions correlated with IQGAP3 expression, we performed analyses based on the Gene Ontology (GO) and Kyoto Encyclopedia of Genes and Genomes (KEGG) database. For the subsequent evaluation and visual representation of the outcomes, we relied on several R packages including “clusterProfiler,” “org.Hs.eg.db,” “enrichplot,” and “ggplot2.” Gene Set Enrichment Analysis (GSEA) was performed to comprehensively evaluate the biological processes associated with IQGAP3 in glioma. Based on the MSigDB Collections gene set database, significantly enriched pathways were identified using nominal (NOM) *p*-values, false discovery rate (FDR) q-values, and normalized enrichment scores (NES). The results were visualized using R packages “ggplot2,” and “gridExtra”.

### Screening of small-molecule drugs targeting IQGAP3

The top 10 overlapping genes from IQGAP3 differential expression and correlation analysis were uploaded to the “query” module of the Cmap online tool (https://clue.io/query) to predict compounds that could potentially treat glioma by targeting IQGAP3. Additionally, the protein crystal structure of IQGAP3 (PDB: 3ISU) was obtained from the PDB database (https://www.rcsb.org/), and the sdf format of target drug structures was retrieved from the PubChem database (https://pubchem.ncbi.nlm.nih.gov/). Molecular docking preparations, including hydrogenation, amino acid modification, energy optimization, and adjustment of force field parameters, were conducted using AutoDock Vina software (http://vina.scripps.edu/). Molecular docking was performed using the vina module within the pyrx software, with the Affinity (kcal/mol) value representing the binding strength between the ligand and the protein. Lower binding affinity indicates a more stable ligand-receptor interaction. Finally, visualization analysis was conducted using PyMOL software.

### Cell culture

The human glioma cell lines SHG-44, CRT, and U251, along with the human brain microvascular endothelial cell line HBMEC, were generously supplied by the Cell Resource Center at the Institute of Life Sciences, Chinese Academy of Medical Sciences. These cell lines were cultivated in DMEM medium supplemented with 10% FBS and were maintained in suitable conditions (37°C, 5% CO_2_).

### Transfection

The plasmid plko.1-CMV-copGFP-PURO-NC (provided by Qingke Biotech, Beijing, China) was used as the vector to construct specific IQGAP3 shRNA plasmids. The shRNA target sequences were 5′-CTCAGTGTGGTACGCAGATTT-3′ (shIQGAP3-1) and 5′-GCAGCTGTTCTTGCCATCAAT-3′ (shIQGAP3-2). Prior to transfection, healthy U251 cells were seeded in six-well plates and divided into control groups (sh-NC) and experimental groups (sh-IQGAP3-1, sh-IQGAP3-2). The cells were allowed to grow overnight to reach 80%–90% confluency before transfection. The transfection procedure was executed utilizing the GenJet^TM^ Plus *in vitro* DNA transfection reagent (SignaGen, USA) in accordance with the prescribed guidelines of the manufacturer. The evaluation of transfection efficiency was carried out through visual assessment of the expression of green fluorescent protein (GFP) originating from the plasmid vector under microscopic observation, with a typical observed efficiency surpassing 70%.

### Quantitative real-time PCR (RT-qPCR)

Conforming to the guidelines provided by the manufacturer, total RNA extraction was conducted utilizing the TRIzol reagent (R401-01, Vazyme, China). This extracted RNA was subsequently transcribed into complementary DNA (cDNA) employing the HiScript II Q RT SuperMix (R223-01, Vazyme, China). The reverse transcription quantitative polymerase chain reaction (RT-qPCR) assay was executed using the Taq Pro Universal SYBR RT-qPCR Master Mix (Q712-02, Vazyme, China). The primer sequences were as follows: IQGAP3 (forward, 5′-GACTACTACAGCCAGTACATCC-3′ and reverse, 5′-CTGATAGTGAAGCTGAAATCGC-3′); PLK1 (forward, 5′-GGCAACCTTTTCCTGAATGA-3′ and reverse, 5′-AATGGACCACACATCCACCT-3′); PI3K (forward, 5′-TCAACCATGACTGTGTGCCA-3′ and reverse, 5′-CCATCAGCATCAAATTGGGCA-3′); AKT (forward, 5′-GGCAAGGGCACTTTCGG-3′ and reverse, 5′-CGGGACAGGTGGAAGAACA-3′); GAPDH (forward, 5′-GGCCTCCAAGGAGTAAGACC-3′ and reverse, 5′-AGGGGTCTACATGGCAACTG-3′). The outcomes were computed employing the 2^−ΔΔCT^ approach, a commonly utilized method in quantitative analysis. All experimental assays were meticulously conducted in triplicate to ensure consistency and reproducibility.

### Western blot assay (WB)

Total protein extraction from cells was performed using RIPA lysis buffer (P0013B, Beyotime, China), followed by determination of protein concentration using a BCA kit (P0010, Beyotime, China). Subsequently, proteins were separated through 10% SDS-PAGE (PG112, Epizyme, China) and transferred to a PVDF membrane, which was then blocked for 1 h with blocking liquid. The PVDF membranes underwent overnight incubation at 4°C with primary antibodies specifically targeting IQGAP3 (DF4389, Affinity, China), PLK1 (A2548, ABclonal, China), p-PI3K (AF3242, Affinity, China), PI3K (4249, CST, USA), p-AKT (4060, CST, USA), AKT (2920, CST, USA), and GAPDH (AC001, ABclonal, China) proteins. Subsequent to a single wash in 3x TBS, the membranes underwent an additional incubation step with HRP-conjugated goat anti-rabbit IgG secondary antibodies (ab6721, Abcam, GBR) for a duration of 1 h, conducted at room temperature. Detection of the blot’s signal was achieved employing the ChemiDoc chemiluminescence system (BIO-RAD, USA).

### EdU assay

The impact of IQGAP3 knockdown on U251 cell proliferation was evaluated using the EdU assay, following the protocols outlined in the EdU cell proliferation kit (C0071S, Beyotime, China). U251 cells were initially incubated in the EdU solution and subsequently fixed using paraformaldehyde (4%) before staining with the click additive solution. Finally, fluorescent images were acquired through employment of fluorescent microscope (Zeiss, Jena, Germany).

### Cell invasion migration assay

The Transwell kit (CLS3397, Corning, USA) was engaged according to the provided guidelines. The lower compartment of the chamber was loaded with medium supplemented with 10% FBS, while the upper compartment hosted 5 × 10^3^ cells suspended in 100 μl of FBS-free medium. Following this, the assembled chambers were introduced into a fresh 24-well plate and incubated at 37°C for a span of 48 h. Upon completion of the incubation period, the medium from the chambers was removed, and the cellular content on the chambers was gently abraded using a cotton swab. Subsequently, the cells that had migrated or invaded into the lower chamber were immobilized utilizing a 4% paraformaldehyde solution, and subsequently, they were stained using a 0.5% crystal violet solution after 24 h of additional culture. Ultimately, cell counting was performed using an inverted light microscope.

### Statistical analysis

The Wilcoxon test was employed for the purpose of juxtaposing the expression levels of IQGAP3 within distinct groups. Pearson correlation analysis was chosen as the methodology to delve into the identification of co-expressed genes that exhibited a connection with IQGAP3 in the context of gliomas. To thoroughly evaluate the ramifications of IQGAP3 on the prognosis of glioma patients, as well as its potential diagnostic significance, a combination of Kaplan-Meier and Cox regression analyses was undertaken.

The rigor of experimentation was upheld through the independent replication of all assays for a total of three iterations. Furthermore, acquired data underwent a normalization process. The subsequent analysis and representation of outcomes were achieved employing software tools such as R 4.0.2, SPSS 22.0, and GraphPad Prism 7.0. Statistical significance was deduced by adopting a two-sided *p*-value threshold of <0.05 for all the conducted statistical assessments.

## Results

### Abnormally high expression of IQGAP3 in gliomas

**Figure 1:**
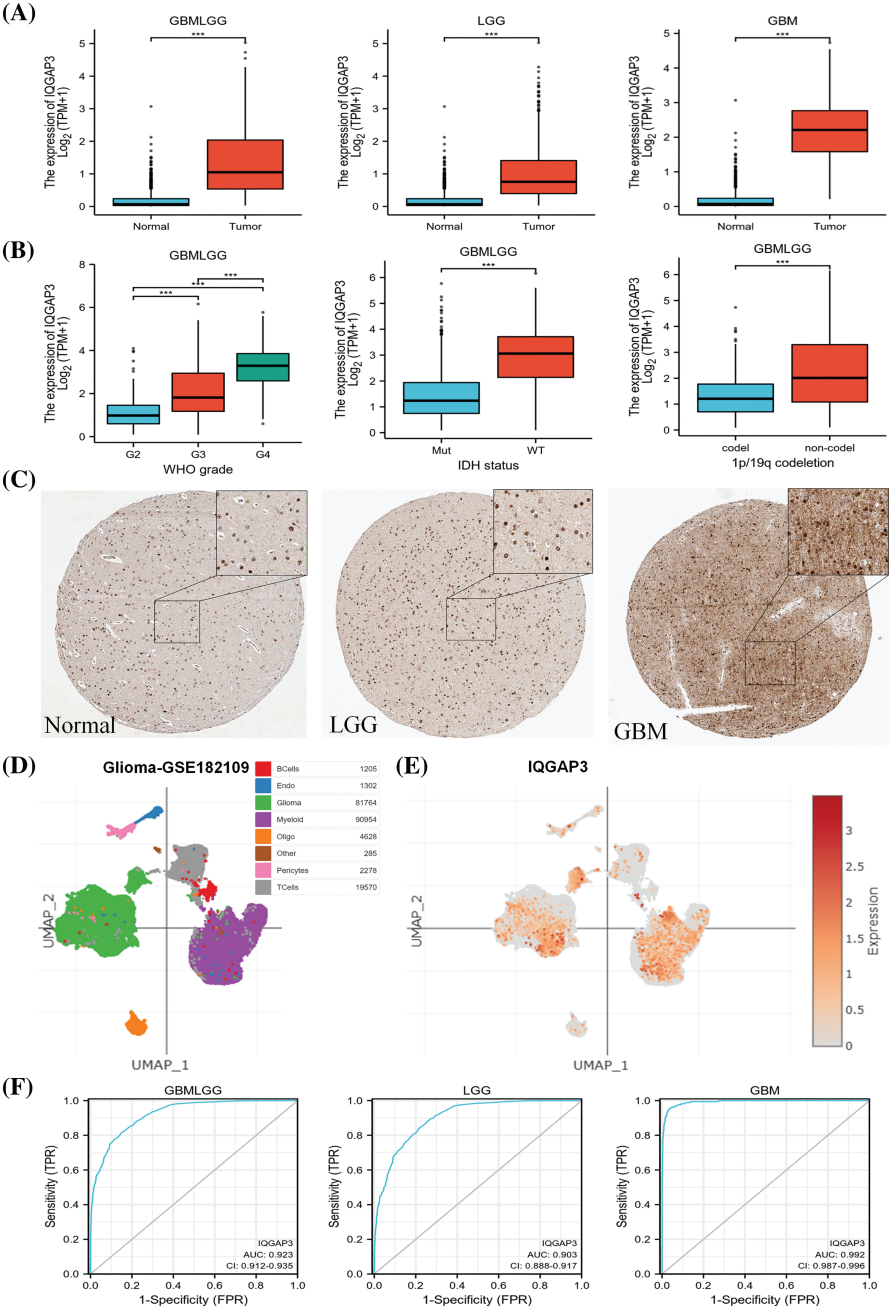
Expression and diagnostic performance of IQGAP3 in glioma. (A) The mRNA expression levels of IQGAP3 in GBMLGG, LGG, GBM, and normal brain tissue samples based on TCGA data, respectively. (B) Differential expression of IQGAP3 in different grades of WHO glioma, the relationship between IQGAP3 expression and IDH mutation, 1p/19q co-deletion in glioma. (C) Protein expression levels of IQGAP3 in gliomas were analyzed using immunohistochemical images from the HPA database. It should be noted that the database does not provide magnification and size information for IHC images. Therefore, we regret that it is not possible to label the scale bars. (D) The tSNE plots of various glioma cell types in the GSE182109 dataset. (E) IQGAP3 expression in different cell types. (F) AUC of ROC curves verified the diagnostic performance of IQGAP3 in glioma with high diagnostic accuracy (AUC: 0.9–1.0). ***p < 0.001.

We analyzed the mRNA expression of IQGAP3 in 689 glioma specimens from the TCGA database and 1152 normal specimens from the TCGA database. Our findings revealed a significant upregulation of IQGAP3 in glioma specimens (*p* < 0.001) (Fig. 1A). Further analysis of IQGAP3 mRNA expression in glioma specimens categorized according to WHO grading (G2: 224 cases, G3: 243 cases, G4: 163 cases) in the TCGA database showed a progressive increase in IQGAP3 expression with the malignant grade of gliomas (*p* < 0.001), in addition, IQGAP3 expression was elevated in the wild-type IDH state as well as in glioma types without 1p/19q co-deletion (Fig. 1B). Immunohistochemical images of IQGAP3 in glioma samples were retrieved from the HPA database. The results displayed positive nuclear staining for IQGAP3 in both high-grade and low-grade gliomas (with some cytoplasmic and membranous expression). The expression of IQGAP3 was stronger in high-grade gliomas, with expression levels exceeding 75%, whereas it exhibited moderate intensity in low-grade gliomas, with expression levels greater than 50%. In normal brain cortical tissue, IQGAP3 staining was relatively low, with levels below 25% (Fig. 1C). To investigate the expression of IQGAP3 at the single-cell level, we utilized the single-cell portal database. Fig. 1D shows the t-SNE plot of different cell types in the GSE182109 dataset, with IQGAP3 being widely expressed in glioma tissue, especially in glioma cells and myeloid cells (Fig. 1E). This indicates a close association between IQGAP3 and glioma. Additionally, myeloid cells are a key immune cell component in the brain glioma microenvironment, related to the pro-tumor progression and immune suppression of glioma [18,19]. IQGAP3 may further promote the progression of glioma through myeloid cells. Next, we used ROC curves to evaluate the diagnostic value of IQGAP3 in glioma. In the GBMLGG, GBM, and LGG datasets, the area under the curve (AUC) was 0.923, 0.903, and 0.992, respectively (Fig. 1F). Our results indicate that compared to non-tumor tissue, IQGAP3 has higher diagnostic accuracy in glioma.

### High IQGAP3 expression suggests poor prognosis for glioma patients

**Figure 2:**
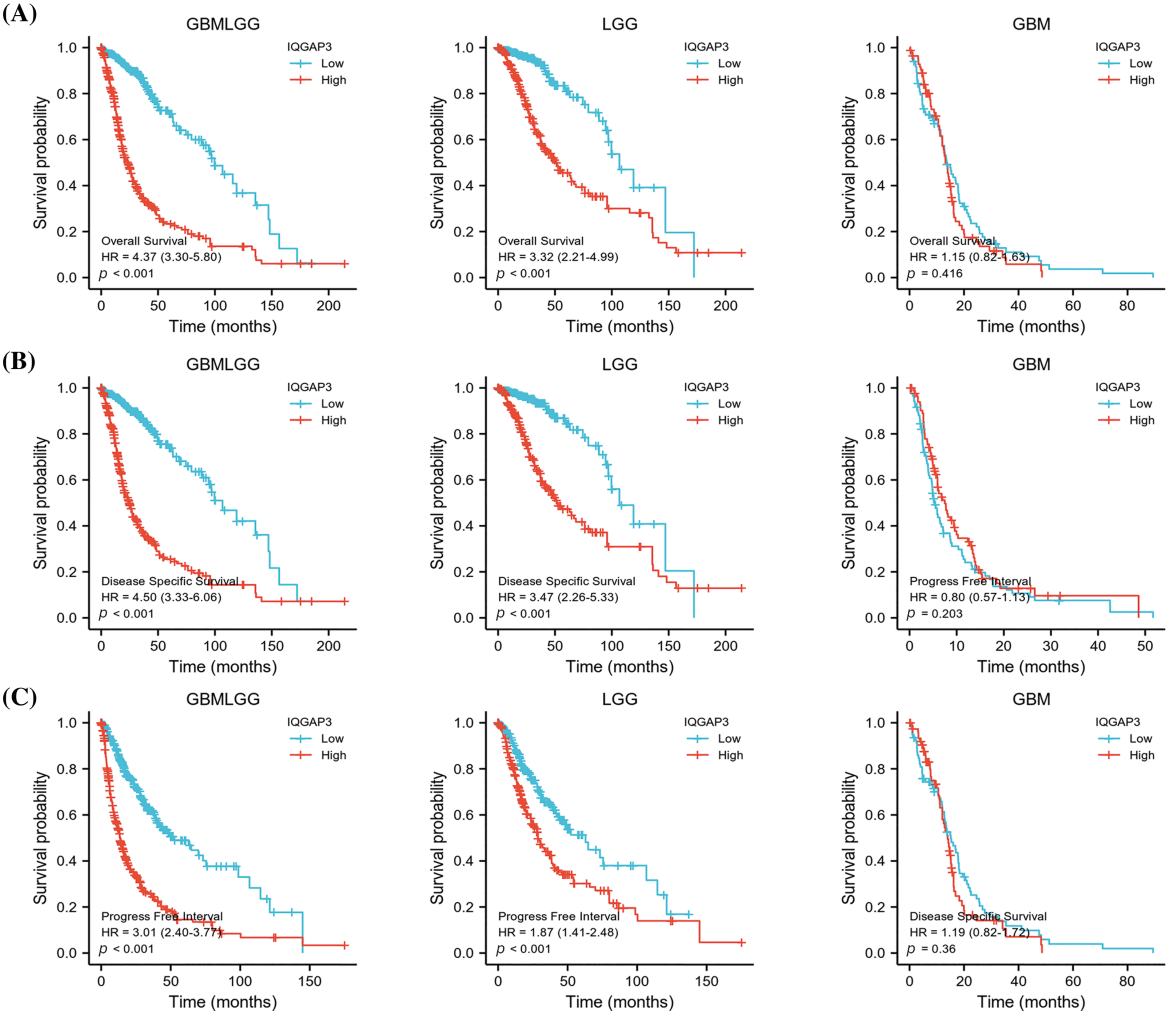
Association of IQGAP3 expression level with glioma prognosis. (A–C) Survival curves of IQGAP3 expression in glioma in relation to OS (overall survival), DSS (disease-specific survival), and PFI (progression-free survival), respectively.

In order to assess the potential impact of IQGAP3 on the survival outcomes of individuals diagnosed with glioma, a comprehensive analysis was conducted utilizing the Kaplan-Meier survival curve methodology. This assessment was facilitated through the utilization of RNA sequencing data procured from the TCGA database. The resultant findings consistently revealed a noteworthy trend: heightened IQGAP3 expression correlated with an unfavorable prognosis in the context of glioma patients, encompassing diverse dimensions of survival including OS (Fig. 2A), DSS (Fig. 2B), and PFI (Fig. 2C). However, for GBM patients, the survival curves did not show a significant prognostic effect.

### IQGAP3 may serve as an independent prognostic factor for glioma patients

To explore the potential correlations between IQGAP3 expression and pertinent clinical-pathological parameters in glioma patients, a Chi-square test was meticulously executed within both the TCGA-GBM and TCGA-LGG cohorts. The subsequent analysis revealed compelling insights: IQGAP3 expression demonstrated a statistically significant linkage with diverse clinical-pathological variables. This encompassed parameters such as WHO grade, IDH status, 1p/19q codeletion, primary therapy outcome, age, histological type, as well as events corresponding to OS, DSS, and PFI (*p* < 0.001). Nevertheless, it is worth noting that no statistically significant association was observed between IQGAP3 expression and gender (*p* = 0.319) (Table 1).

**Table 1:** Correlation between IQGAP3 expression and clinical parameters in patients with GBMLGG in the TCGA-GBMLGG cohort

Characteristic	IQGAP3 (Low)	IQGAP3 (High)	*p*-value
*n*	348	348	
WHO grade, *n* (%)			<0.001
G2	182 (28.7%)	42 (6.6%)	
G3	113 (17.8%)	130 (20.5%)	
G4	15 (2.4%)	153 (24.1%)	
IDH status, *n* (%)			<0.001
WT	43 (6.3%)	203 (29.6%)	
Mut	301 (43.9%)	139 (20.3%)	
1p/19q codeletion, *n* (%)			<0.001
codel	127 (18.4%)	44 (6.4%)	
non-codel	220 (31.9%)	298 (43.3%)	
Primary therapy outcome, *n* (%)			<0.001
PD	46 (10%)	66 (14.3%)	
SD	91 (19.7%)	56 (12.1%)	
PR	49 (10.6%)	15 (3.2%)	
CR	99 (21.4%)	40 (8.7%)	
Gender, *n* (%)			0.319
Female	156 (22.4%)	142 (20.4%)	
Male	192 (27.6%)	206 (29.6%)	
Age, *n* (%)			<0.001
<=60	315 (45.3%)	238 (34.2%)	
>60	33 (4.7%)	110 (15.8%)	
Histological type, *n* (%)			<0.001
Astrocytoma	116 (16.7%)	79 (11.4%)	
Glioblastoma	15 (2.2%)	153 (22%)	
Oligoastrocytoma	84 (12.1%)	50 (7.2%)	
Oligodendroglioma	133 (19.1%)	66 (9.5%)	
OS event, *n* (%)			<0.001
Alive	285 (40.9%)	139 (20%)	
Dead	63 (9.1%)	209 (30%)	
DSS event, *n* (%)			<0.001
Alive	286 (42.4%)	145 (21.5%)	
Dead	56 (8.3%)	188 (27.9%)	
PFI event, *n* (%)			<0.001
Alive	235 (33.8%)	115 (16.5%)	
Dead	113 (16.2%)	233 (33.5%)	
Age, median (IQR)	39 (32, 51)	53 (39, 63)	<0.001

A comprehensive assessment was undertaken through univariate and multivariate Cox regression analyses, aiming to elucidate the interplay between IQGAP3 expression and the OS trends within both cohorts. The outcomes of the univariate Cox regression analysis unveiled compelling associations, as IQGAP3 expression levels demonstrated statistically significant correlations with pivotal clinical variables including patient WHO grade, mutation type, clinical efficacy, gender, and age in the context of OS prediction. The multivariate Cox regression analysis revealed that IQGAP3 was an independent prognostic factor for glioma patients. These findings suggest that IQGAP3 expression may serve as a novel prognostic biomarker independent of tumor grade and other prognostic factors (Table 2).

**Table 2:** Univariate and multivariate analyses of IQGAP3 expression associated with OS in GBMLGG patients in the TCGA-GBMLGG cohort

Characteristics	Total (N)	Univariate analysis		Multivariate analysis	
		Hazard ratio (95% CI)	*p*-value	Hazard ratio (95% CI)	*p*-value
WHO grade	634				
G2	223	Reference			
G3	243	2.999 (2.007–4.480)	**<0.001**	1.605 (0.967–2.663)	0.067
G4	168	18.615 (12.460–27.812)	**<0.001**	4.565 (1.344–15.508)	**0.015**
IDH status	685				
WT	246	Reference			
Mut	439	0.117 (0.090–0.152)	**<0.001**	0.455 (0.264–0.785)	**0.005**
1p/19q codeletion	688				
codel	170	Reference			
non-codel	518	4.428 (2.885–6.799)	**<0.001**	1.062 (0.553–2.042)	0.856
Gender	695				
Female	297	Reference			
Male	398	1.262 (0.988–1.610)	0.062	1.751 (1.117–2.745)	**0.015**
Age	695				
<=60	552	Reference			
>60	143	4.668 (3.598–6.056)	**<0.001**	4.030 (2.421–6.706)	**<0.001**
Primary therapy outcome	461				
PD	112	Reference			
SD	147	0.440 (0.294–0.658)	**<0.001**	0.342 (0.204–0.571)	**<0.001**
PR	64	0.170 (0.074–0.391)	**<0.001**	0.188 (0.066–0.532)	**0.002**
CR	138	0.133 (0.064–0.278)	**<0.001**	0.160 (0.074–0.347)	**<0.001**
Histological type	695				
Astrocytoma	195	Reference			
Glioblastoma	168	6.791 (4.932–9.352)	**<0.001**		
Oligoastrocytoma	134	0.657 (0.419–1.031)	0.068	1.120 (0.645–1.944)	0.687
Oligodendroglioma	198	0.580 (0.395–0.853)	**0.006**	0.620 (0.356–1.081)	0.092
IQGAP3	695				
Low	348	Reference			
High	347	4.372 (3.296–5.801)	**<0.001**	1.418 (0.899–2.236)	0.033

### The impact of IQGAP3 gene mutations on glioma

**Figure 3:**
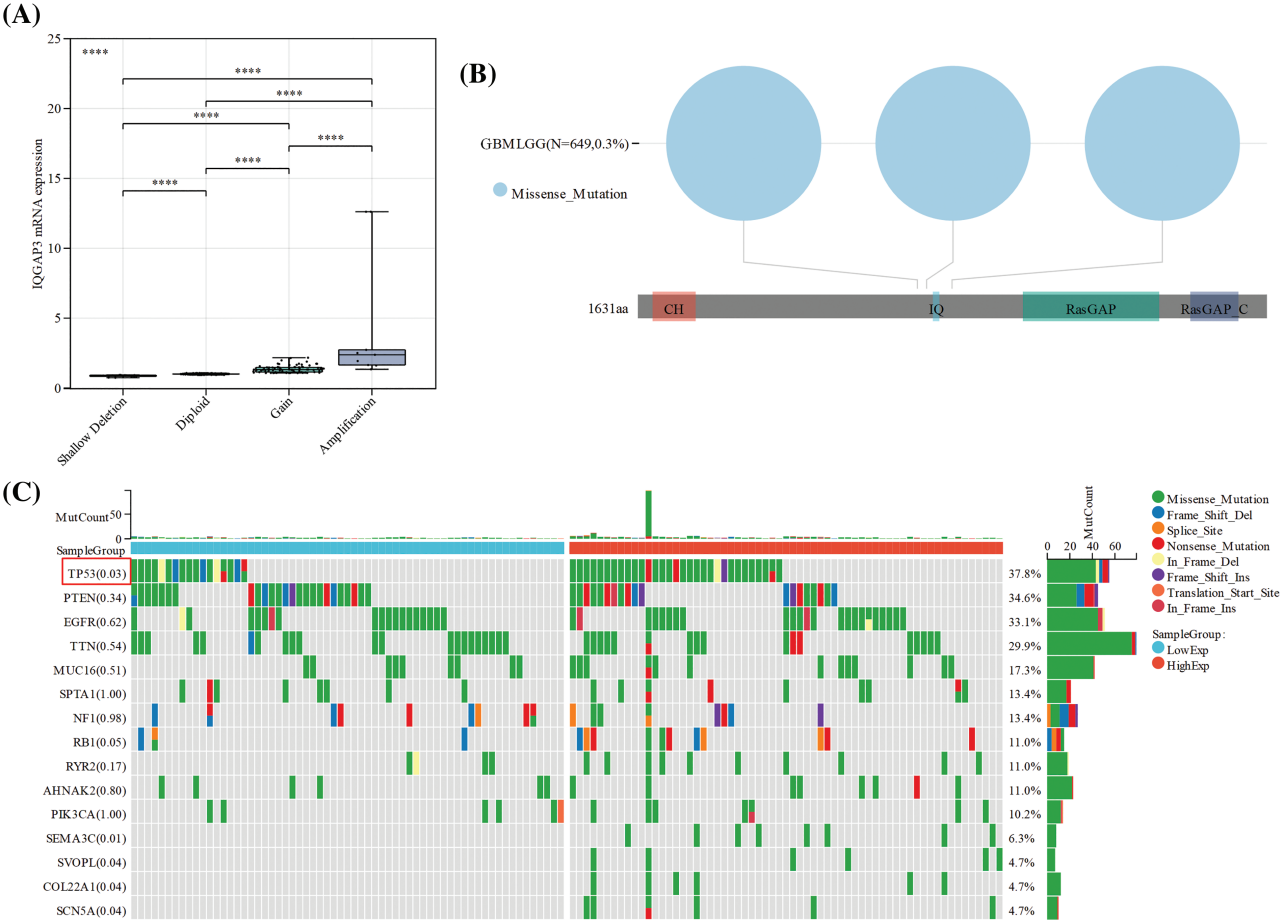
Association of genetic alterations in the IQGAP3 gene with gliomas. (A) IQGAP3 mRNA expression in different copy number alteration types. (B) Analysis of IQGAP3 mutation sites in gliomas by cBioPortal tool. (C) Detection of differential somatic mutations in IQGAP3 in gliomas, gene mutation landscape showing only the top 15 genes with the highest mutation rates.

Genetic mutations, deletions, and amplifications of oncogenes or tumor suppressor genes have been linked to the proliferation and advancement of various tumor types [20]. We delved into the intricate relationship between the expression of IQGAP3 mRNA and its copy number alterations using the cbioportal database. The data revealed that most patients exhibited IQGAP3 copy number amplifications and copy number gains (low-level amplifications) (Fig. 3A). Moreover, an examination of the cBioPortal database revealed the prevalence of missense mutations as the principal category among IQGAP3 gene mutations within gliomas, as depicted in Fig. 3B. Subsequently, a comprehensive investigation was performed to elucidate the interplay between IQGAP3 expression and distinct genomic attributes, notably somatic mutations, within the TCGA glioma dataset. Utilizing the IQGAP3 expression profiles in glioma specimens, patients were classified into cohorts denoting heightened and diminished expression levels. The high-expression group included 140 GBM and 190 LGG samples, while the low-expression group comprised 13 GBM and 319 LGG samples. The results showed that the mutation frequency of the TP53 (*p* = 0.03) gene was significantly increased in high IQGAP3-expressing samples (Fig. 3C), suggesting a potential functional interaction between IQGAP3 and TP53.

### The relationship between IQGAP3 expression and tumor immune infiltration

**Figure 4:**
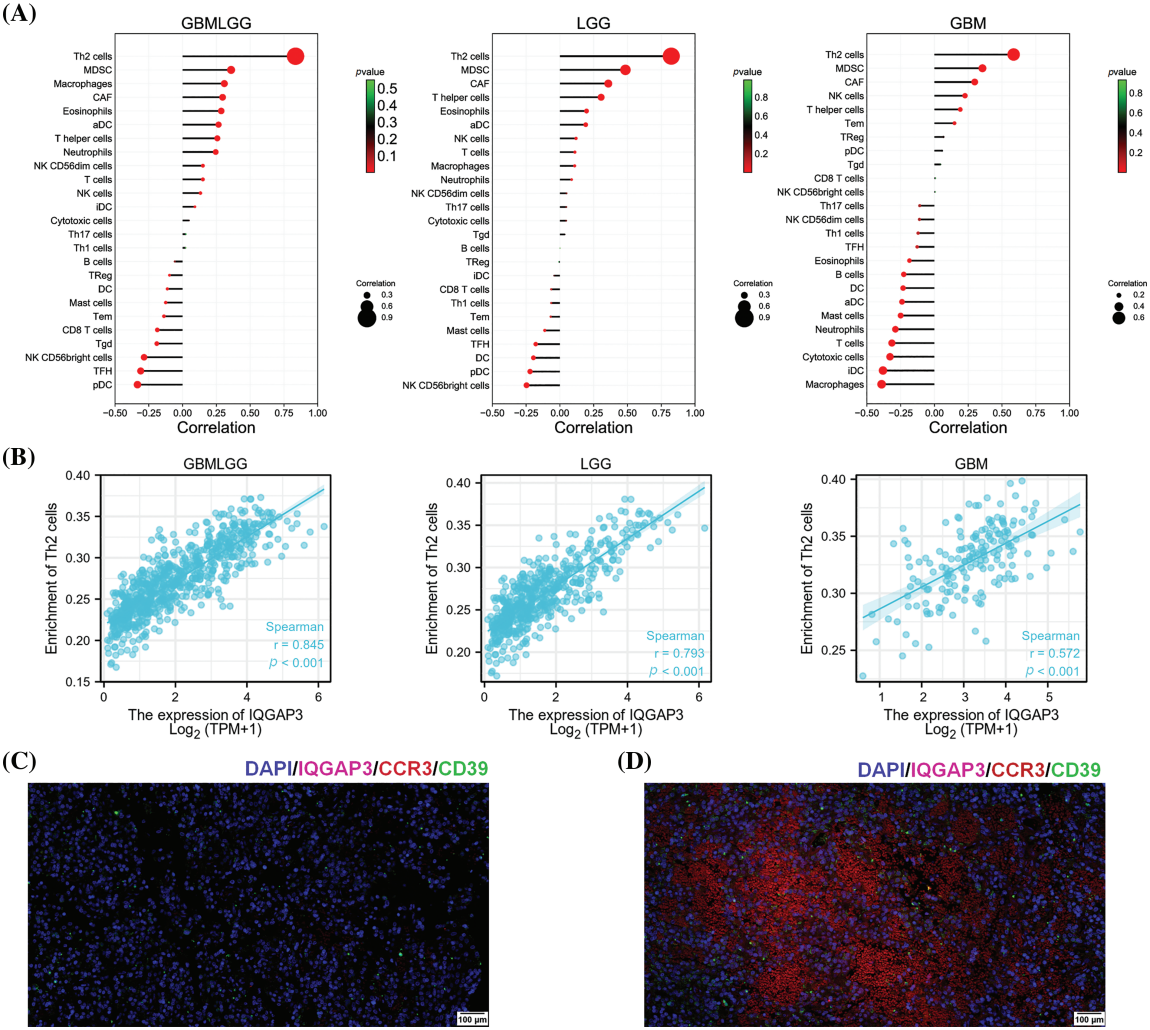
Relationship between IQGAP3 expression and tumor immune infiltration. (A) Correlation between IQGAP3 and 25 immune infiltrating cells in glioma. The circles’ size and the bars’ height represent the degree of correlation, and the color’s shade represents the *p*-value’s magnitude. (B) Scatter plot in glioma demonstrating the relationship of Th2 cells significantly correlated with IQGAP3. (C) The representative fluorescence images of paracarcinoma tissue regions. (D) The representative fluorescence images of tumor tissue regions. DAPI (blue), IQGAP3 (fuchsia), CCR3 (red), CD39 (green).

Tumor-infiltrating immune cells (ICs) play a key role in suppressing/promoting tumor growth [21]. Based on the average expression of IQGAP3, we categorized all glioma samples into high-expression and low-expression groups. The proportions of 25 distinct immune cell types within the GBMLGG, GBM, and LGG groups were estimated utilizing the ssGSEA method (Suppl. Fig. 1). Subsequently, the Spearman correlation method was employed to evaluate the correlation between the expression of IQGAP3 and the levels of infiltration of 25 immune cell types. As shown in Fig. 4A, in the GBMLGG group, IQGAP3 expression positively correlated with immune cells such as Th2 cells, myeloid-derived suppressor cells (MDSC), macrophages, cancer associated fibroblast cells (CAF), eosinophils, activated dendritic cells (aDC), T helper cells, Neutrophils and Neutrophils, and negatively correlated with plasmacytoid dendritic cells (pDC), NK CD56bright cells, and T follicular helper (TFH) cells (|Correlation| > 0.2, *p* < 0.05). Within the context of the LGG cohort, IQGAP3 expression positively correlated with Th2 cells, MDSC, CAF, T helper cells, and antigen-presenting dendritic cells (aDC), and negatively correlated with NK CD56bright cells. Conversely, in the GBM subset, IQGAP3 expression demonstrated favorable correlations with Th2 cells, MDSC, CAF, and NK cells, juxtaposed with adverse correlations involving Macrophages, immature dendritic cells (iDC), Cytotoxic cells, T cells, conventional dendritic cells (DC), Neutrophils, Mast cells, aDC, Eosinophils, and B cells.

Scatter plots demonstrated the strongest correlation between IQGAP3 expression and Th2 cells in gliomas, including GBMLGG (r = 0.845, *p* < 0.001), GBM (r = 0.572, *p* < 0.001), and LGG (r = 0.793, *p* < 0.001) (Fig. 4B). To further validate the role of IQGAP3 in the tumor immune microenvironment, we employed a multi-color fluorescence immunohistochemistry approach to assess the differential expression of IQGAP3, CCR3 (a surface marker for Th2 cells), and CD39 (a surface marker for MDSCs) in human glioblastoma tissue compared to corresponding adjacent non-tumor tissue. The results showed that compared with adjacent non-tumor tissues (Fig. 4C), IQGAP3 was more widely expressed in glioblastoma (Fig. 4D). In addition, when comparing the high-expression group and the low-expression group of IQGAP3, the infiltration levels of Th2 cells and MDSCs cells were higher in the high-expression group, and pictures of each channel of immunofluorescence are provided in Suppl. Fig. 2. Our experimental results provide more evidence that IQGAP3 is significantly overexpressed in the glioblastoma tissue, and the immune infiltration levels of Th2 cells and MDSCs cells are higher in the glioblastoma tissue, highlighting the close relationship between IQGAP3 and the immune-suppressive microenvironment.

### The effect of IQGAP3 on the efficacy of tumor immunotherapy

**Figure 5:**
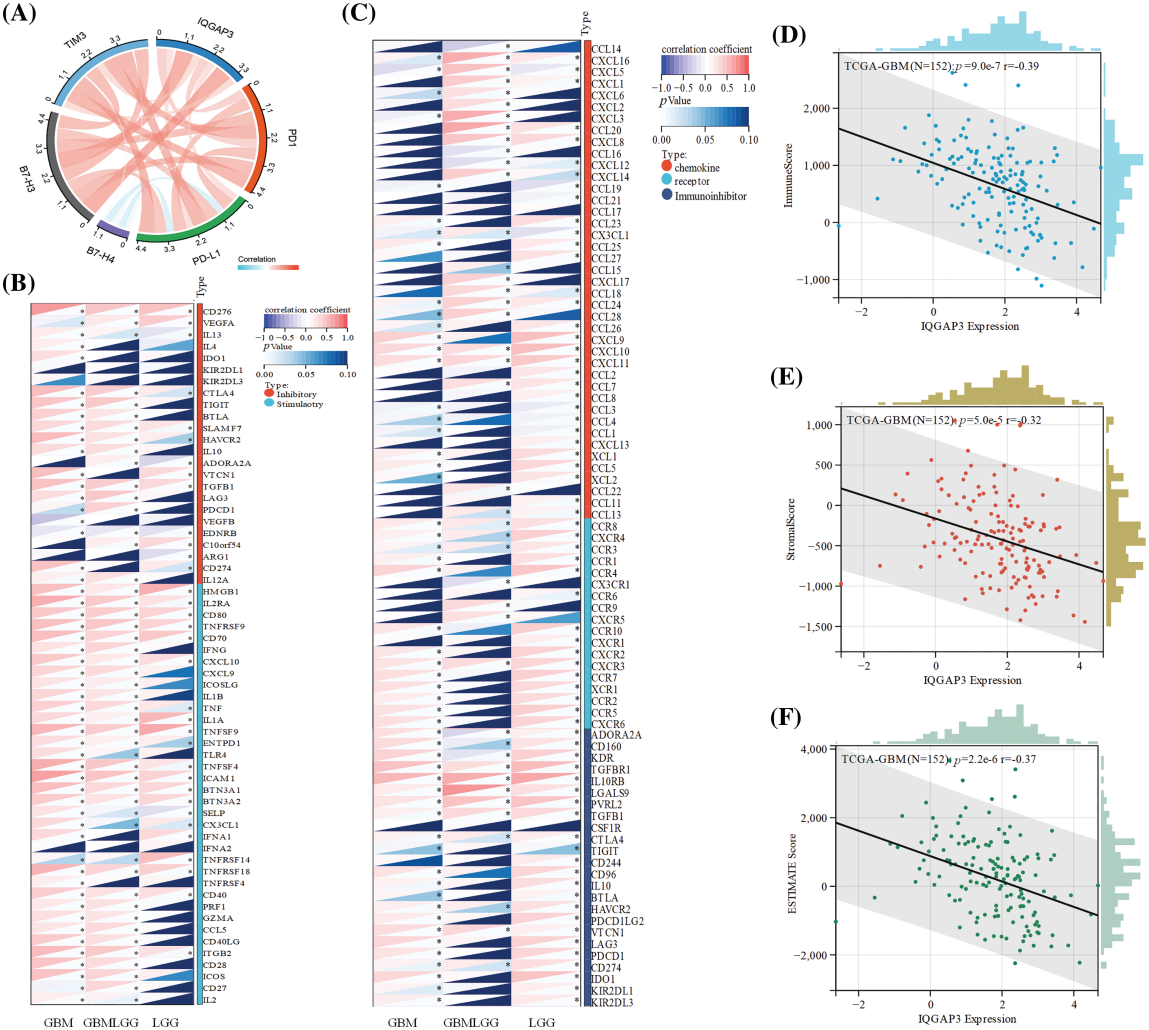
Correlation of IQGAP3 with immunotherapy indicators. (A) Association between IQGAP3 expression and immune checkpoints. The bands’ thickness and the color’s shade represent the correlation’s magnitude. (B–C) Association of IQGAP3 expression with ICP genes and immune-related genes. Red indicates a positive correlation, and blue indicates a negative correlation. * Represents statistically significant. (D–F) Relationship between IQGAP3 expression and immune scores, stromal scores, and ESTIMATE scores.

Cancer cells possess the capacity to activate different immune checkpoints, a realm where immune checkpoint inhibitors have ushered in noteworthy advancements in cancer therapy [22]. In order to elucidate the interconnection between IQGAP3 and immune checkpoints, Spearman analysis was employed to scrutinize the correlation existing between IQGAP3 and pivotal constituents of immune checkpoints. The results showed a positive correlation between IQGAP3 and PD1, PD-L1, B7-H3 and TIM3 (Fig. 5A), thereby signifying that an overexpression of IQGAP3 triggers activation within the PD1, PD-L1, B7-H3, and TIM3 pathways. Furthermore, a more extensive exploration was conducted concerning the nexus between IQGAP3 and genes associated with immune checkpoints, alongside genes related to immunity. Evidently, IQGAP3 expression exhibited affirmative correlations with a majority of immune checkpoint genes (Fig. 5B) and immune-related genes (Fig. 5C). ESTIMATE score is an important indicator reflecting the status of the tumor immune microenvironment and tumor purity [23]. Therefore, we assessed the correlation between immune, stromal, and ESTIMATE scores with IQGAP3 expression levels in glioma. The results showed that IQGAP3 expression was negatively correlated with all scores, including −0.39 for the immune score (Fig. 5D), −0.32 for the stromal score (Fig. 5E), and −0.37 for the ESTIMATE score (Fig. 5F). This suggests that IQGAP3 expression is associated with reduced immune and stromal cell infiltration in glioma, leading to higher tumor purity.

### IQGAP3-related biological processes

**Figure 6:**
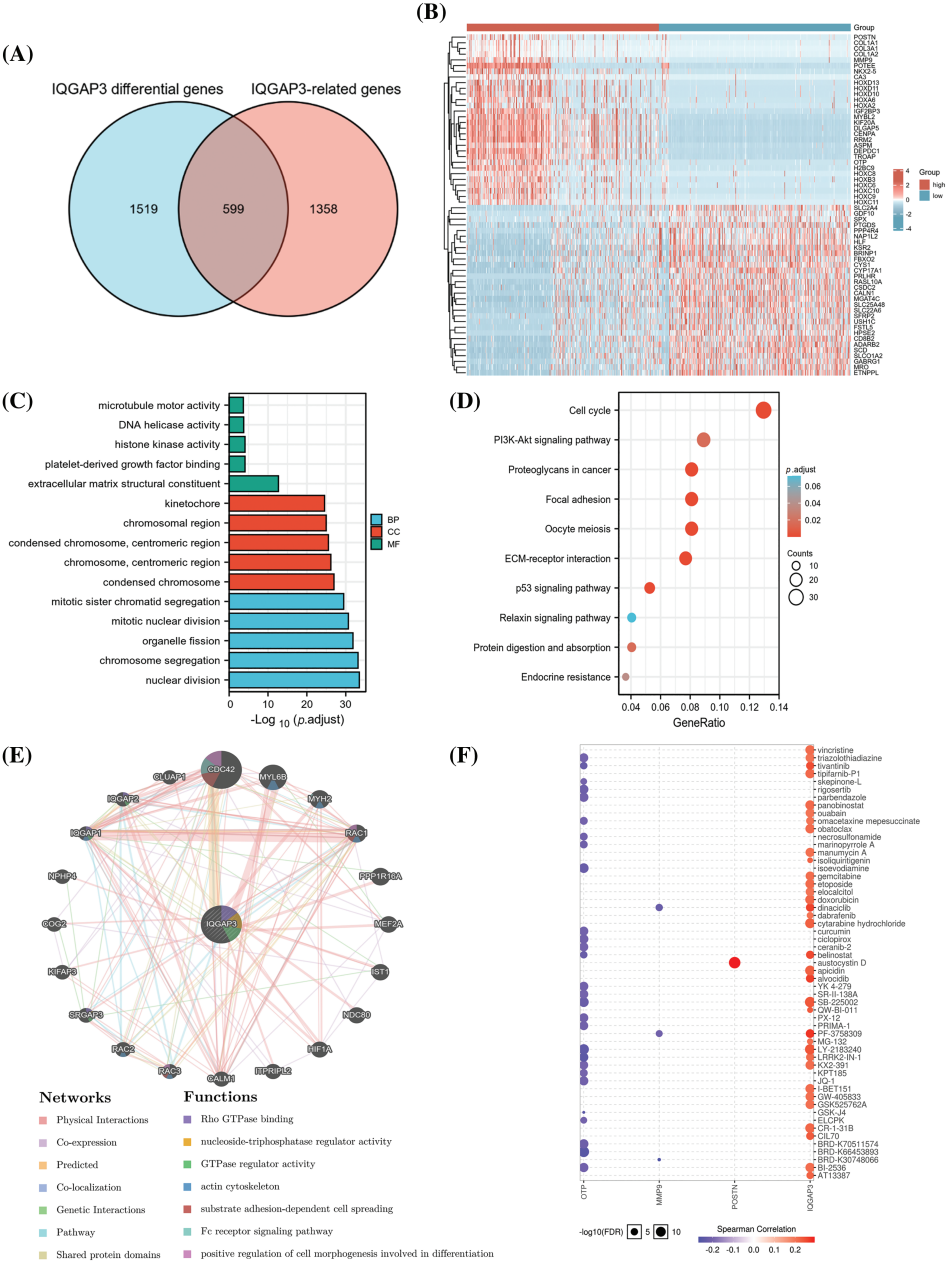
Functional enrichment analysis of differential genes associated with IQGAP3 in glioma. (A) Wayne plot demonstrating the overlap between IQGAP3 differentially expressed genes and IQGAP3-related genes for 599 genes. (B) Heat map demonstrating the top 30 and bottom 30 differentially expressed genes associated with IQGAP3 in the low and high IQGAP3 expression groups in gliomas. (C–D) GO and KEGG enrichment analysis of 599 genes IQGAP3-associated differential genes. (E) Protein-protein interaction (PPI) network of IQGAP3 (GeneMANIA). (F) Drug sensitivity analysis in IQGAP3-related gene set of Drug Sensitivity in glioma. The Color indicated Spearman’s correlation coefficient and the circle size represented the False Discovery Rate (FDR) value.

We identified IQGAP3-related differentially expressed genes (DEGs) between the IQGAP3 low-expression and high-expression groups, resulting in a total of 2,118 DEGs (Suppl. Table 1). Using the Pearson method in glioma samples, we extracted genes correlated with IQGAP3 expression and filtered out 1,957 IQGAP3-related genes (Suppl. Table 2). Combining the above DEGs and IQGAP3-related genes, we conducted Venn analysis and obtained 599 IQGAP3-related differentially expressed genes (RDEGs) (Fig. 6A). These 599 RDEGs were annotated using the annotation file downloaded from the TCGA database, and the heatmap displayed the top 30 upregulated and top 30 downregulated genes (Fig. 6B).

To explore the potential functions of the selected 599 RDEGs, we conducted GO and KEGG pathway analyses. GO enrichment analysis revealed that in the biological process (BP), the genes were mainly enriched in nuclear division, chromosome segregation, and mitosis; in the cellular component (CC), they were mainly enriched in the chromosomal region and centromeric region; and in the molecular function (MF), they were mainly enriched in extracellular matrix structural constituent, platelet-derived growth factor, and histone kinase activity (Fig. 6C). KEGG pathway analysis indicated that the RDEGs were mainly involved in pathways such as Cell cycle, PI3K-Akt signaling pathway, Proteoglycans in cancer, Focal adhesion, Oocyte meiosis, ECM-receptor interaction, and p53 signaling pathway (Fig. 6D). These findings imply a pivotal role for IQGAP3 in the trajectory of tumor development through its modulation of the cell cycle and cancer-associated signaling pathways. The Protein-Protein Interaction (PPI) network constructed by GeneMANIA further illustrated the biological functions among IQGAP3-related genes. The results show that the IQGAP3 gene set is mainly involved in biological processes such as Rho GTPase binding, nucleoside-triphosphatase regulator activity, GTPase regulator activity, and actin cytoskeleton (Fig. 6E). In a further exploration, an analysis of genomic patterns governing cancer drug sensitivity unveiled a significant correlation between the heightened expression of IQGAP3 and small molecules and anticancer agents. This observation lays a potential foundation for the prospect of drug-targeted therapeutic approaches (Fig. 6F).

**Figure 7:**
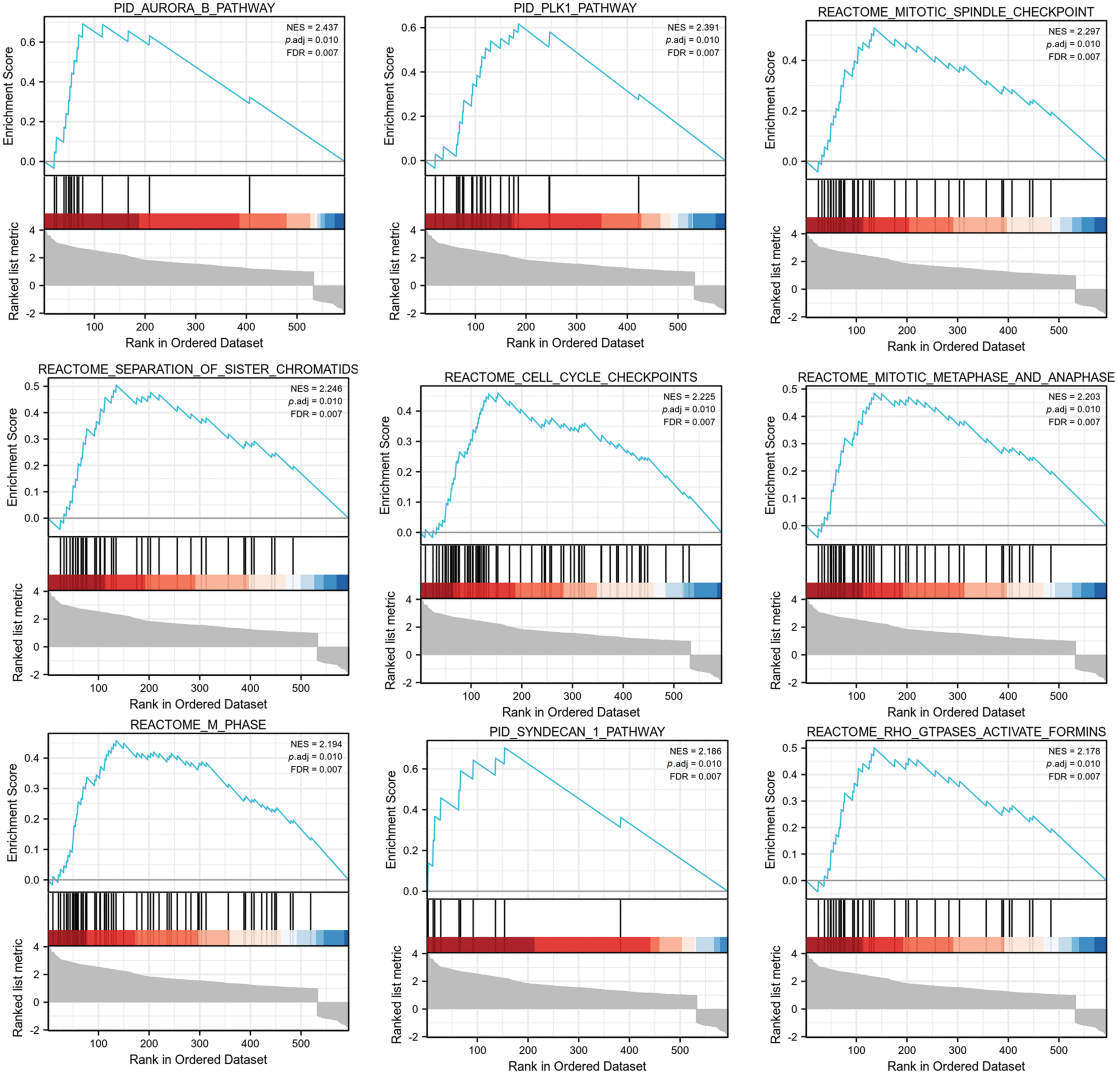
GSEA analysis of IQGAP3-associated differential genes in glioma. The pathway of IQGAP3 high expression group enrichment is shown in the figure.

Furthermore, we performed GSEA on the IQGAP3-related differentially expressed genes. The GSEA data showed that there were 9 pathways significantly different between the IQGAP3 high-expression and low-expression groups, including Aurora-B pathway, PLK1 pathway, mitotic spindle checkpoint, sister chromatid segregation, cell cycle checkpoint, mitotic spindle checkpoint, m phase, syncytial 1 pathway, RHO GTPASES activation (Fig. 7).

### Screening of potential small-molecule anti-glioma drugs targeting IQGAP3

**Figure 8:**
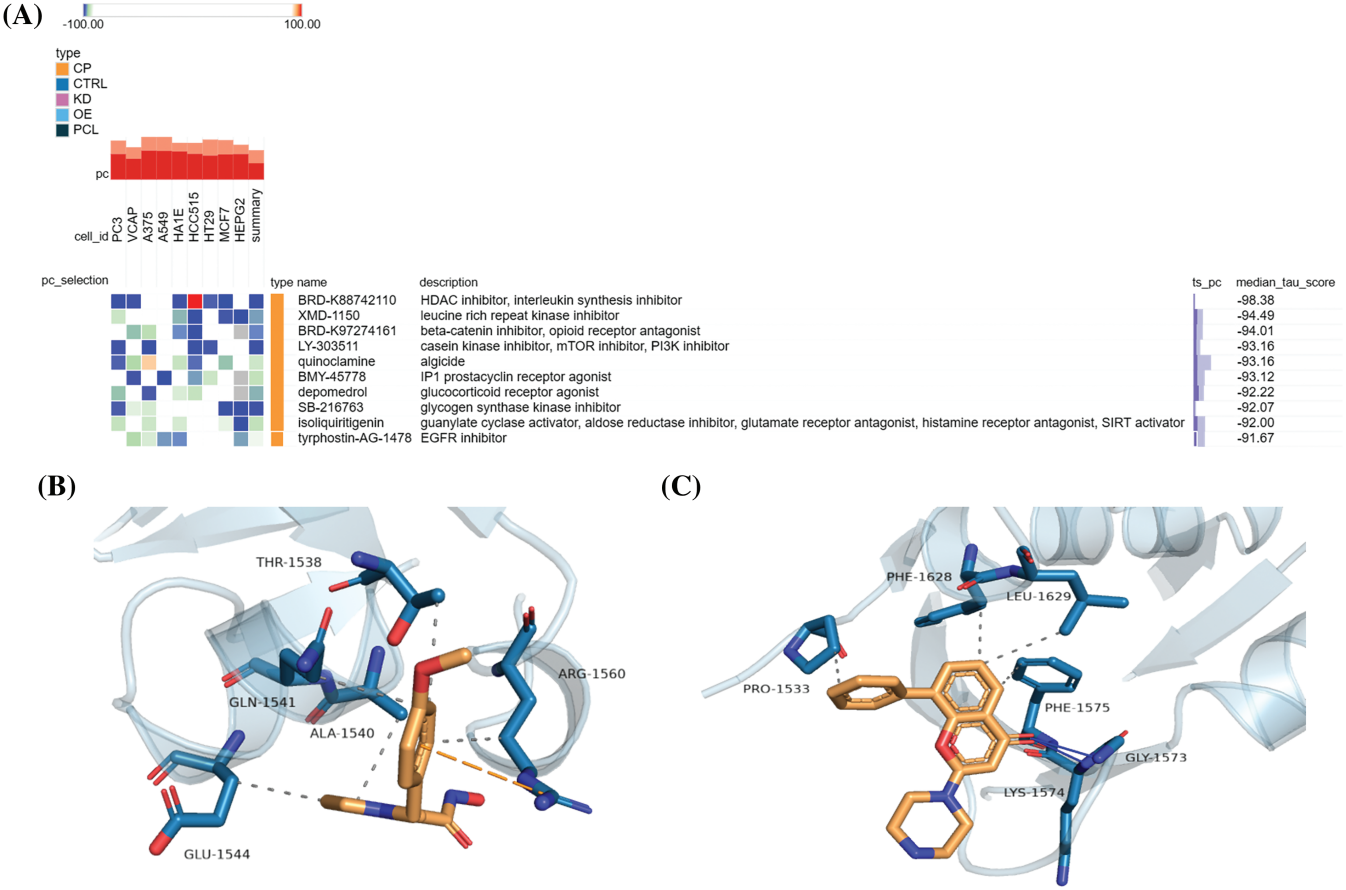
Anti-glioma small molecule drugs targeting IQGAP3. (A) Promising compounds against gliomas were screened by the CMap platform, and the heatmap shows the top 10 negatively correlated small molecules, with blue indicating negative correlation and red indicating positive correlation. (B) The results of molecular docking simulations of IQGAP3 with compound BRD-K88742110. (C) The results of molecular docking simulations of IQGAP3 with compound LY-303511. Solid blue lines indicate hydrogen-bonded hydrophilic interactions and dashed lines indicate hydrophobic interactions.

To identify promising small-molecule drugs targeting IQGAP3 in glioma, we uploaded the top 10 overlapping genes from IQGAP3 differential expression and correlation analysis to the Cmap online tool. By comparing gene expression profile data, drugs highly correlated with the disease were identified, and the top 10 negatively correlated compounds were displayed based on median scores (Fig. 8A). These compounds were found to reverse gene alterations in different tumor cell lines significantly. Notably, BRD-K88742110 (an HDAC inhibitor and interleukin synthesis inhibitor), LY-303511 (a casein kinase inhibitor, mTOR inhibitor, and PI3K inhibitor), and SB-216763 (a glycogen synthase kinase inhibitor) were among the compounds negatively correlated with the cell lines in the heatmap. Previous research has shown that SB-216763 can significantly inhibit the proliferation of glioma cells and induce apoptosis. Additionally, it inhibits the growth of mouse brain transplant tumors and enhances mouse survival rates [24]. In contrast, BRD-K88742110 and LY-303511 have yet to be extensively studied in glioma.

Therefore, we conducted molecular docking, and the results indicated that BRD-K88742110 and LY-303511 could bind to the active pocket of the IQGAP3 protein and interact with surrounding amino acid residues. For BRD-K88742110, IQGAP3 forms hydrophobic interactions with the target protein’s residues GLN-1541, ALA-1540, THR-1538, ARG-1560, and GLU-1544, with a docking binding energy of −6.3 kcal/mol (Fig. 8B). For LY-303511, IQGAP3 forms hydrogen bond hydrophilic interactions with the target protein’s residues GLY-1573 and LYS-1574, as well as hydrophobic interactions with PRO-1533, PHE-1628, LEU-1629, and PHE-1575, with a docking binding energy of −6.6 kcal/mol (Fig. 8C). In summary, BRD-K88742110 and LY-303511 may be potential IQGAP3-targeting drugs, offering new directions for small-molecule drug development in glioma.

### Knockdown of IQGAP3 can suppress the proliferation, invasion, and migration of glioma cells

**Figure 9:**
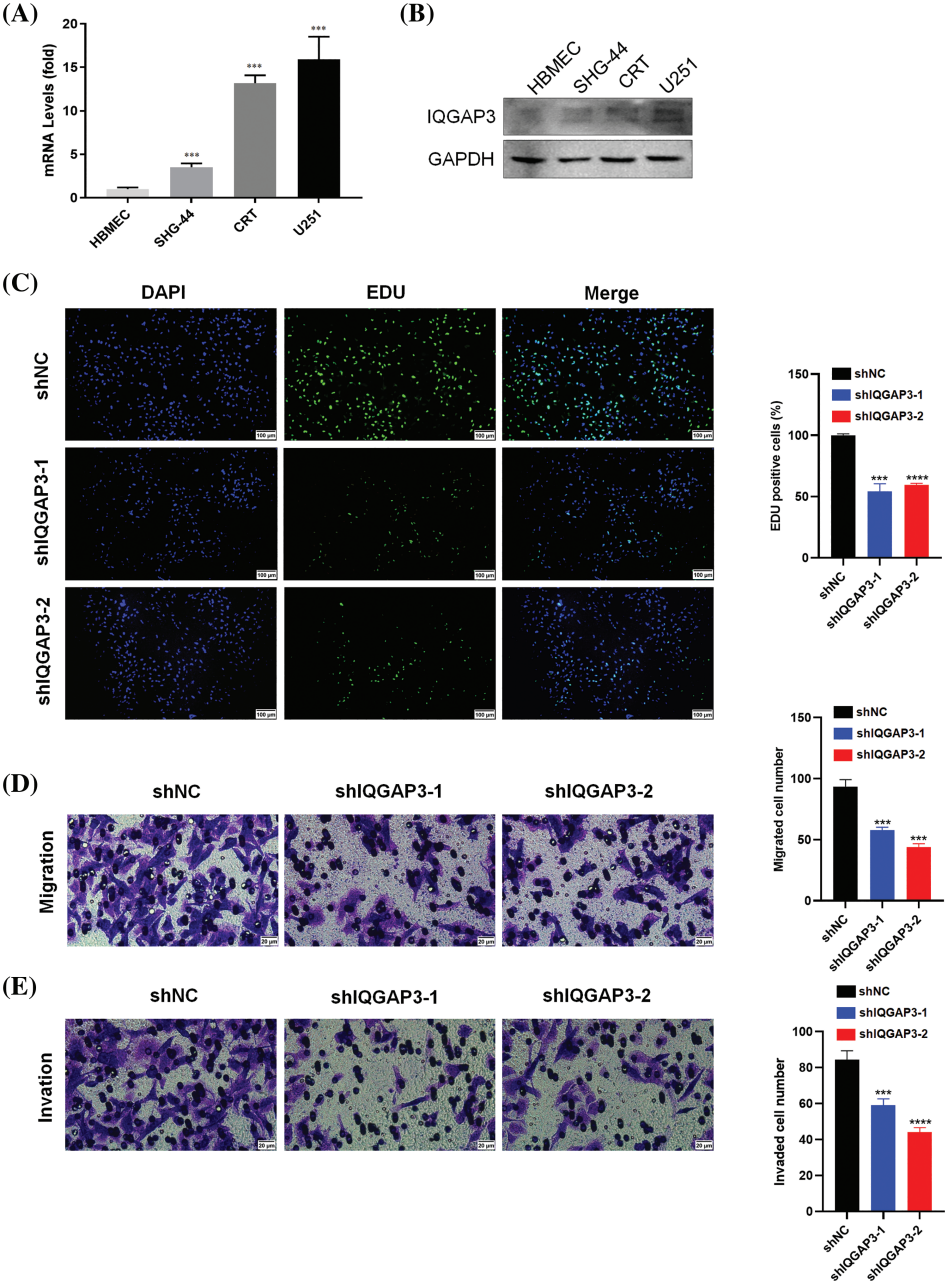
Effect of IQGAP3 knockdown on glioma cells. (A) The mRNA expression of IQGAP3 in human brain microvascular endothelial cell lines and human glioma cell lines. (B) The protein expression of IQGAP3 in human brain microvascular endothelial cell lines and human glioma cell lines. (C) EdU assay outcomes for cellular proliferative potential. (D) The results of Transwell assay for cell migration. (E) The results of Transwell assay for cell invasion. *p < 0.05, **p < 0.01, ***p < 0.001, ****p < 0.0001.

The expression of IQGAP3 in human glioma cell lines, SHG-44, CRT, U251, and human brain microvascular endothelial cell line HBMEC, was analyzed through RT-qPCR. The outcomes unequivocally exhibited an upregulation of IQGAP3 expression in SHG-44, CRT, and U251 cells when compared to normal brain cells (Fig. 9A), a finding further confirmed by Western blotting analysis (Fig. 9B). Among them, U251 cells with the highest expression of IQGAP3 were selected for subsequent investigations. In an effort to delve deeper into the influence of IQGAP3 on glioma cell proliferation, invasion, and migration capabilities, U251 cells were transfected with shRNA targeting IQGAP3.

The EdU assay unveiled a substantial reduction in the population of EdU-positive cells within the shIQGAP3 group, in stark contrast to the shNC group (Fig. 9C). Transwell experiments indicated that knocking down IQGAP3 reduced the number of glioma cells involved in migration (Fig. 9D) and invasion (Fig. 9E). In conclusion, our findings suggest that IQGAP3 knockdown can suppress the progression of glioma cells *in vitro*.

### The potential molecular mechanisms of IQGAP3 in U251 cells

**Figure 10:**
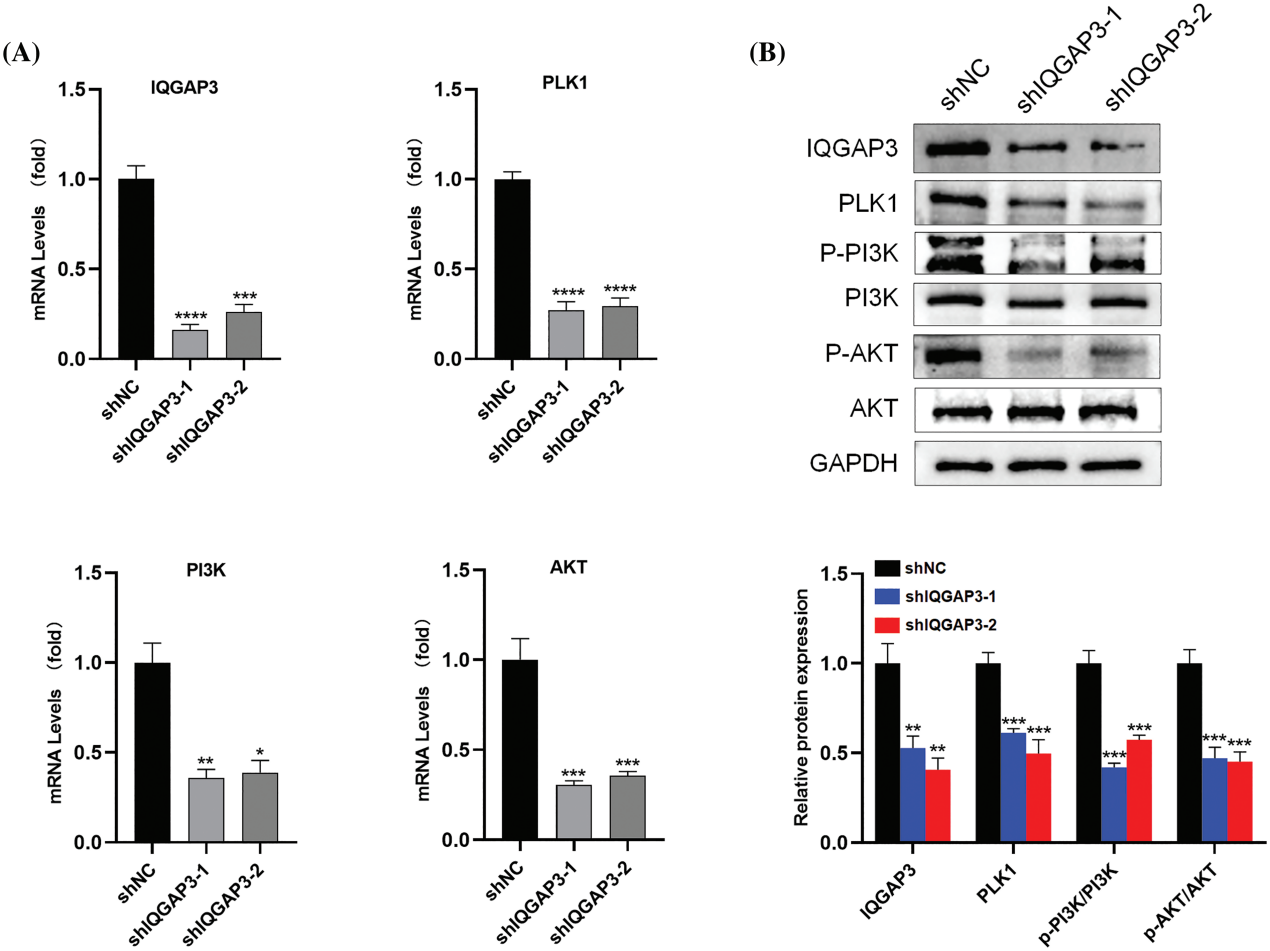
The downstream molecular mechanisms of IQGAP3 in U251 cells. (A) The mRNA expression levels of IQGAP3 PLK1, PI3K, AKT in shNC and shIQGAP3 groups of U251 cells. (B) The relative protein expression levels of IQGAP3 PLK1, PI3K, p-PI3K, AKT and p-AKT in shNC and shIQGAP3 groups of U251 cells. *p < 0.05, **p < 0.01, ***p < 0.001, ****p < 0.0001.

Based on previous bioinformatics analysis, we identified that IQGAP3 regulates several crucial signaling pathways in glioma. Among them, IQGAP3 is significantly enriched in pathways associated with PLK1 and PI3K/AKT. However, the molecular mechanisms of IQGAP3 in relation to the PLK1/PI3K/AKT pathway in glioma have not been explored. In pursuit of understanding this phenomenon, we conducted an investigation into the expression levels of molecules pertinent to the PLK1/PI3K/AKT pathway in U251 cells transfected with shNC and shIQGAP3 using RT-qPCR and Western blotting. The outcomes unmistakably showcased that the suppression of IQGAP3 in U251 cells precipitated a marked decrease in the expression of IQGAP3 itself. This was subsequently accompanied by the dampening of PLK1 expression and a reduction in the phosphorylation status of PI3K and AKT when compared to the control group (Fig. 10A), findings that are in harmony with the Western blotting results (Fig. 10B).

## Discussion

Glioma stands as the predominant malignant neoplasm affecting the brain, constituting approximately 40% to 50% of the overall incidence of intracranial tumors [25]. Due to its high invasiveness and recurrence rate, the treatment of glioma still faces significant challenges. Therefore, identifying key drivers of glioma could potentially offer novel therapeutic strategies such as inhibition of oncogenic signaling pathways, targeted therapies, and immunotherapy [26].

IQGAP3 is crucial for cell cycle, genome stability and tumor stem cell potential [27]. Growing evidence suggests that IQGAP3 is a biogenetic or prognostic marker of malignancy that promotes migration, invasion, and drug resistance in many human malignancies [28], such as gastric cancer [29], clear cell renal cell carcinoma [13], bladder cancer [30], and liver cancer [31]. This suggests that IQGAP3 may be an important oncogene. However, its mechanism of action in glioma has not been reported to date.

This study is the first to investigate IQGAP3 as a biomarker in glioma. Through bioinformatics analysis, we explored the diagnostic, prognostic, genetic alterations, immune infiltration levels, and biological functions of IQGAP3 in glioma. In addition, a series of experimental validations were performed to evaluate the influence of IQGAP3 on various aspects of glioma biology, including proliferation, migration, and key signaling pathways. The findings demonstrate a significant correlation between elevated expression levels of IQGAP3 and higher WHO grades, wild-type IDH status, as well as the absence of co-deletion of 1p/19q. IDH1 status is an important molecular feature of glioma, with wild-type IDH frequently found in primary GBMs, while IDH mutations are observed in GBMs arising from LGGs [32]. Previous studies have shown that wild-type IDH and higher WHO grades (G3 or G4) are associated with poor outcomes in glioma [33]. Furthermore, compared to 1p/19q co-deletion, glioma patients with non-co-deletion of 1p/19q have shorter overall survival [34]. Kaplan-Meier survival curve analysis and Cox regression analysis further confirmed these findings. Therefore, overexpression of IQGAP3 in glioma is significantly associated with poor prognosis in patients and serves as an independent prognostic factor for glioma patients.

The epigenetic remodeling in gliomas is recognized as a pivotal factor in the development of these tumors, as well as in their response to immunotherapy and the determination of clinical characteristics [35]. Therefore, this research delved into the potential mechanisms through which IQGAP3 exerts its influence on gliomas, with a specific focus on genetic and epigenetic alterations. The findings demonstrated a significant correlation between the upregulation of IQGAP3 mRNA and the increased copy number and amplification of IQGAP3. Studies have indicated that gene copy number amplification may foster intratumoral genetic heterogeneity and expedite tumor evolution [36]. Implying that genetic modifications instigated by IQGAP3 in gliomas may further hasten glioma progression. Furthermore, somatic mutations in pivotal genes can contribute to the conversion of healthy cells into malignant cancer cells [37], and these somatic mutations within cancer cells can undermine immune evasion and hinder the efficacy of therapeutic interventions [38]. We found that the frequency of TP53 gene mutations was significantly increased in the high expression group of IQGAP3 compared to the low expression group. The tumor suppressor p53 (TP53) is a well-known oncogene, and TP53 mutations are frequently observed in a wide range of cancers [39]. In gliomas, TP53 mutations result in a genetic signature associated with the progression of GBM, the inflammatory response, and shorter overall survival [40]. The high expression of IQGAP3 in gliomas induced more TP53 mutations, suggesting that IQGAP3 is a promising candidate as an important biomarker for gliomas.

The tumor immune microenvironment (TIME) assumes a pivotal role in the pathogenesis of cancer [41]. Improving TIME is beneficial for cancer immunotherapy [42]. TIME mainly consists of components such as tumor cells, immune cells, and cytokines [43]. Consequently, an estimation was conducted to determine the relative proportions of 25 distinct immune cell types within glioma, followed by an analysis of the correlation between the expression of IQGAP3 and these immune cell populations. The research findings indicate that IQGAP3 is associated with various immune cell types, particularly with CD4 Th2 cells and MDSCs present in all subtypes of gliomas, and the results of mIHC further validate the hypotheses generated from the bioinformatics analysis. Th2 cells are a subtype of CD4+T cells [44]. In cancer, the balance of helper T cells tends to shift from Th1 to Th2 cells, promoting B cells to produce antibodies to evade anti-tumor responses from normal cells [45]. There is substantial evidence suggesting that Th2 immunity promotes cancer incidence, invasion, and metastasis [46]. MDSCs are abundantly recruited within the TME, are known to exert immunosuppressive effects. These cells impede T cell functionality, thereby promoting cancer invasiveness, metastasis, and immune tolerance [47,48]. Furthermore, chemokines and chemokine receptors mediate the transportation of immune cells to the tumor microenvironment [49]. Their expression in malignant tumors determines immune cell activation, angiogenesis, and cancer cell proliferation and metastasis [50]. We discovered a positive correlation between high levels of IQGAP3 expression and chemokines, chemokine receptors, and immune inhibitory genes. Therefore, IQGAP3 may potentially facilitate tumor immune evasion by stimulating the intricate interactions between cellular immune components and immune-infiltrating cells. In summary, IQGAP3 has the potential to exert a significant impact on tumor progression and immune suppression through its modulation of the immune microenvironment within the tumor milieu.

Immune checkpoints (ICPs) are essential immune regulators that maintain immune homeostasis and prevent autoimmune responses [51]. However, in the context of cancer, there is a frequent activation of immune checkpoint pathways, resulting in the disruption of anti-tumor immune responses and promoting the uncontrolled growth and proliferation of cancerous cells [52]. Programmed cell death 1 (PD-1) and programmed death-ligand 1 (PD-L1) are the most extensively studied and recognized inhibitory checkpoint pathways, and their interaction induces the activation of the host immune system through T cell responses, thereby resulting in immune escape of tumor cells [53,54]. Additionally, some emerging immune checkpoints are under investigation, such as T cell immunoglobulin and mucin-domain containing-3 (TIM-3), B7 homolog 3 protein (B7-H3), and B7 homolog 4 protein (B7-H4) [55]. Targeting ICPs (immune checkpoint inhibitors, ICIs) can limit T cell receptor (TCR) signaling [56]. As a result, ICI therapy has brought significant breakthroughs in cancer immunotherapy for patients [57]. Our results showed that IQGAP3 expression was positively correlated with immune checkpoint genes (PD1, PD-L1, B7-H3, TIM3) and most of the immune checkpoint-associated genes in gliomas, suggesting that IQGAP3 may be promising as a new immune checkpoint for gliomas. In addition, IQGAP3 expression was associated with higher glioma purity, which is a key factor affecting the response to immunotherapy and prognosis of patients, and high tumor purity was associated with poor prognosis of patients [58]. These results suggest that IQGAP3 affects the sensitivity of gliomas to ICIs therapy. Targeting IQGAP3 may improve the prognosis of glioma patients and bring new hope for cancer immunotherapy.

To reveal the possible biological role of IQGAP3 in gliomas, we conducted enrichment analysis using 599 RDEGs. The GO enrichment analysis results indicated that IQGAP3 may be involved in biological processes such as cell division and cell cycle regulation. Furthermore, employing KEGG and GSEA enrichment analysis, we successfully identified several signaling pathways that exhibited enrichment in the high IQGAP3 expression group. These pathways encompassed critical cellular processes such as the cell cycle, mitosis, M checkpoint, PI3K/AKT signaling pathway, p53 signaling pathway, Aurora-B pathway, PLK1 pathway, and Rho GTPases activation. According to reports, processes related to the cell cycle, mitosis, and the M checkpoint are associated with cancer cell proliferation [59]. Additionally, IQGAP3 has been established as an essential factor for cell cycle progression and the maintenance of genomic stability [11]. The PI3K/AKT signaling pathway, which governs various cellular processes including cell growth, proliferation, metabolism, angiogenesis, and migration [60], has been implicated in promoting glioma progression [61]. The PI3K/Akt signaling pathway can serve as a therapeutic target for glioma [62]. The p53 pathway undergoes mutations in the majority of cancers and plays a role in the growth, proliferation, and apoptosis of cancer cells, thereby suppressing the progression of glioma [63]. Aurora-B plays a central role in mitosis, and there are reports suggesting that Aurora-B, as an effective oncogene, is overexpressed or amplified in a wide range of human malignancies [64]. Polo-like kinase 1 (PLK1) is involved in regulating DNA repair, cell proliferation, autophagy, apoptosis, and epithelial-to-mesenchymal transition [65]. Emerging research has revealed the frequent overexpression of PLK1 in various cancer types, which correlates with unfavorable prognostic outcomes, rendering it an appealing candidate for cancer treatment [66]. Rho GTPases have been implicated in tumor cell proliferation and survival, exerting a pivotal role in cancer cell invasion and metastasis. Multiple *in vitro* and *in vivo* investigations have consistently demonstrated the tumor-promoting effects associated with activated Rho GTPases [67]. For experimental validation, we selected the most significantly enriched genes related to IQGAP3, specifically PLK1 and the PI3K/AKT pathway. The results demonstrated that IQGAP3 is highly expressed in glioma cell lines and that knocking down IQGAP3 significantly inhibits the proliferation and migration of glioma cells. Furthermore, we observed that knocking down IQGAP3 leads to reduced expression of proteins associated with the PLK1/PI3K/AKT pathway in glioma cells. In summary, IQGAP3 likely plays a crucial role in initiating and progressing glioma through multiple cancer-related pathways, including the PLK1/PI3K/AKT pathway. These enriched genes and pathways are often associated with glioma proliferation and malignancy.

Furthermore, we utilized the Cmap online tool to identify small-molecule drugs targeting IQGAP3 for combating glioma. We also conducted molecular docking simulations for two compounds not previously studied in glioma: BRD-K88742110 and LY-303511. BRD-K88742110 is an experimental HDAC inhibitor. High expression of Histone Deacetylases (HDACs) has been associated with cancer pathology and regulates tumor invasion, cell apoptosis, and angiogenesis [68]. HDAC inhibitors have been demonstrated as effective inducers of growth arrest and cell death in various cancer cells, including glioblastoma [69]. LY-303511 is a casein kinase inhibitor, mTOR inhibitor, and PI3K inhibitor. Studies have shown that LY-303511 enhances the oligomerization of DR5, assembly of the DISC complex, and mitochondrial permeabilization, thereby amplifying tumor necrosis factor-related apoptosis-inducing ligand (TRAIL)-induced apoptosis in cancer cells and potentially increasing the sensitivity of chemotherapy-resistant tumors [70,71]. Hence, these two small-molecule compounds may play significant roles in anti-glioma therapy targeting IQGAP3, supporting personalized treatment approaches and clinical decision-making.

However, this study possesses several limitations that warrant consideration. Firstly, the majority of our findings rely on data analysis, which may introduce a degree of uncertainty regarding the data’s accuracy. Secondly, our research solely offers preliminary evidence regarding the association between IQGAP3 and glioma, necessitating further experimental validation to unravel the precise molecular functions and mechanisms through which IQGAP3 influences the occurrence and progression of glioma. Our future research will encompass a broader range of animal models and clinical samples, employing molecular biology experiments to elucidate further the relationship between IQGAP3, genetic mutations, and cancer signaling pathways. Additionally, the validation of screened targeted small-molecule drugs will be conducted to enhance the potential utility of IQGAP3 as a biomarker for cancer immunity and prognosis.

## Conclusion

In summary, IQGAP3 is highly expressed in glioma to varying degrees and is associated with poor prognosis and several clinical features. Various genetic alterations of IQGAP3 have been identified in glioma, including missense mutations, copy number increases, and amplifications. Additionally, IQGAP3 expression shows significant correlations with tumor immune microenvironment, multiple immune markers, and various cancer-related signaling pathways in glioma. Therefore, it may serve as a potential biomarker for glioma prognosis and immunotherapy response. *In vitro* experiments have confirmed the oncogenic function of IQGAP3 in glioma. Our study suggests that IQGAP3 is a promising prognostic biomarker and a potential predictive factor for immunotherapy sensitivity, providing insights for future efforts in achieving more precise targeted therapies and immunotherapies.

### Highlights

1. IQGAP3 is aberrantly expressed in gliomas and is associated with the clinical characteristics and genetic alterations observed in patients.
2. The overexpression of IQGAP3 is correlated with a poor prognosis in glioma patients and can independently serve as a prognostic factor for glioma patients.
3. IQGAP3 may play a role in promoting tumor progression and immune evasion by influencing the immune microenvironment of the tumor.
4. IQGAP3 can influence the sensitivity of gliomas to immunotherapy. Targeting IQGAP3 might potentially enhance the prognosis of glioma patients, thus offering new prospects for cancer immunotherapy.

## Supplementary Materials

Supplementary Figure 1 The proportion of 24 immune-infiltrating cells in glioma samples from the IQGAP3 high and low expression groups. ns, not significant; *p<0.05; **p<0.05; ***p<0.001.

Supplementary Figure 2 Immunofluorescence images of each channel. (A) Paracancerous tissue region. (B) Tumor tissue region. DAPI (blue), IQGAP3 (fuchsia), CCR3 (red), CD39 (green).

## Data Availability

The source of the raw/processed data needed to reproduce these findings is in the Methods section. All data, models, or code generated or used during the study are available from the corresponding author by request.

## Supplementary Materials

The supplementary material is available online at https://doi.org/10.32604/or.2023.046712.
