# Supplementary figures and images for: IQGAP3 promotes the progression of glioma as an immune and prognostic marker
Source: Oncol Res. 2024 Mar 20;32(4):659–78. doi: 10.32604/or.2023.046712 (PMC10972721; doi:10.32604/or.2023.046712)

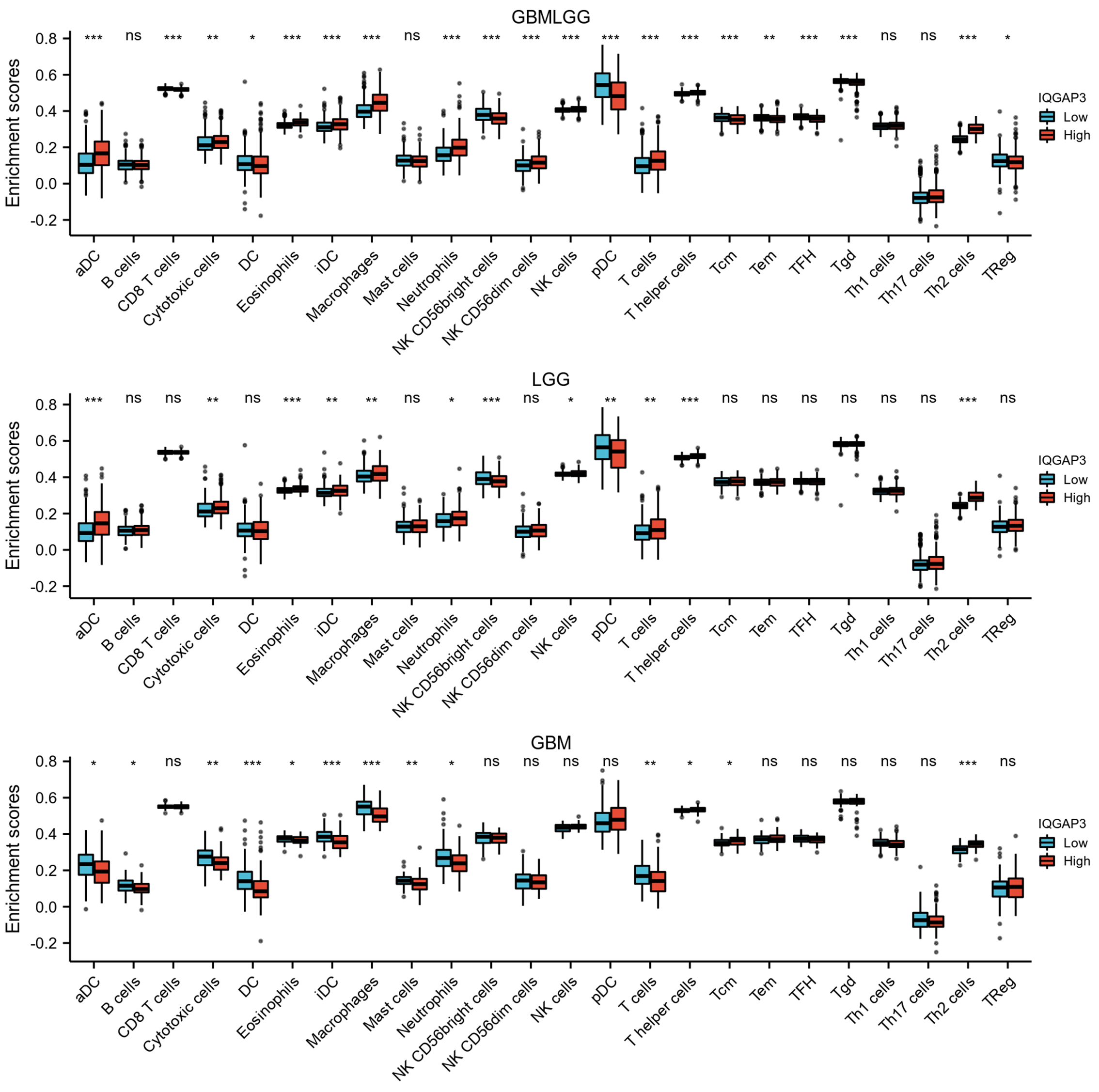

Supplement: Supplementary Figure 1 [file OncolRes-32-46721-s001.tif]

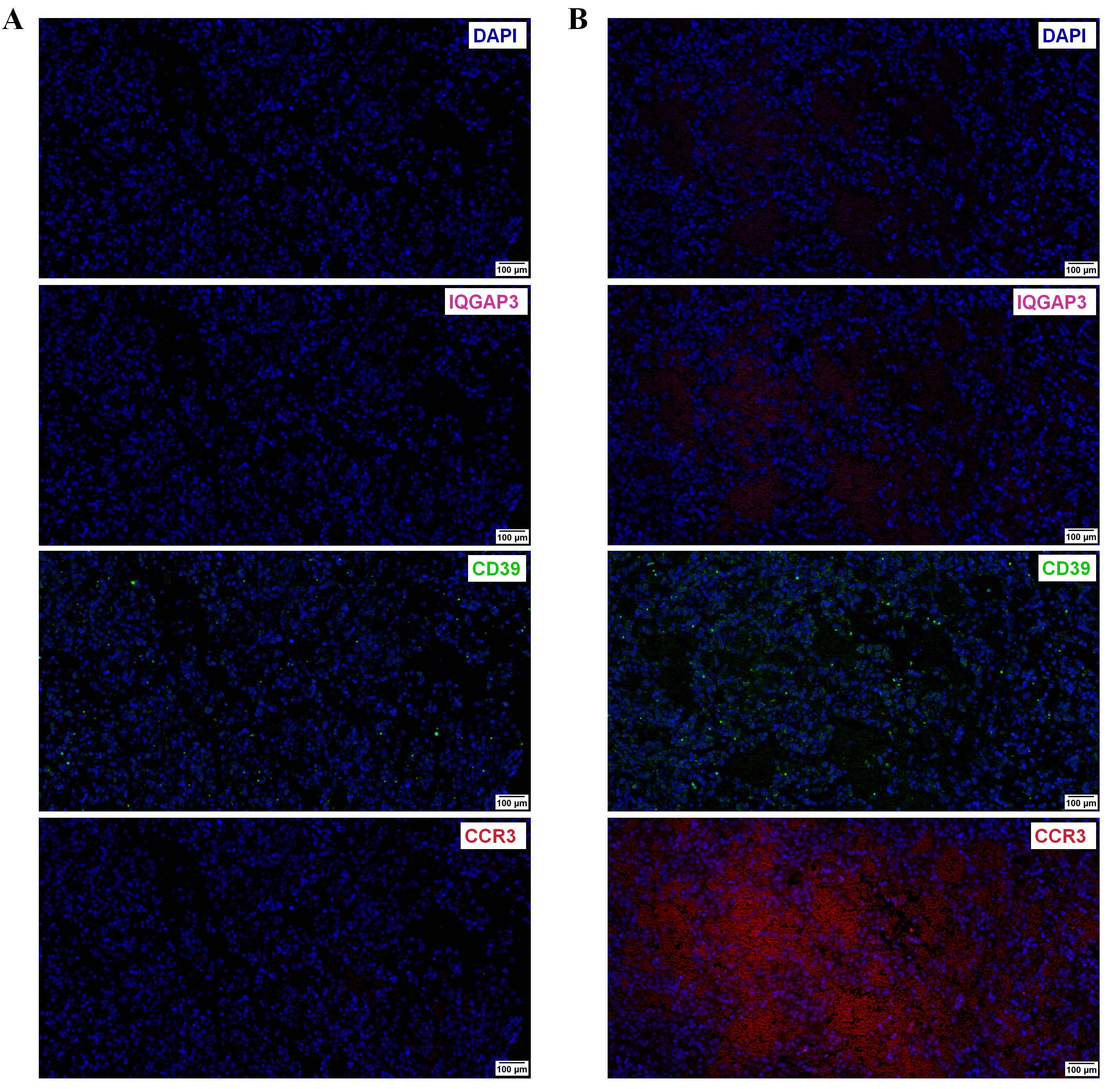

Supplement: Supplementary Figure 2 [file OncolRes-32-46721-s002.tif]
